# Placenta Previa and Pre-Eclampsia: Analyses of 1645 Cases at Medani Maternity Hospital, Sudan

**DOI:** 10.3389/fphys.2013.00032

**Published:** 2013-02-28

**Authors:** Ishag Adam, AbdElrahium D. Haggaz, Omer A. Mirghani, Elhassan M. Elhassan

**Affiliations:** ^1^Faculty of Medicine, University of KhartoumKhartoum, Sudan; ^2^Faculty of Medicine, University of GeizeraMedani, Sudan

**Keywords:** placenta previa, pre-eclampsia, pregnancy, vascular, Sudan

## Abstract

A retrospective case-control study was conducted to investigate the risk factors for pre-eclampsia – including the protective effect of placenta previa – at Medani Maternity Hospital, Sudan. Medical files of the patients during the period 2003–2010 were reviewed for age, parity, education level, prenatal care, placenta previa, and hemoglobin level. Women with pre-eclampsia were the cases, and women with normal pregnancy were the controls. There were 54,339 singleton deliveries and 1765 women with pre-eclampsia in the hospital, giving the incidence of pre-eclampsia of 3.2%. The risk factors for pre-eclampsia were; women with age >35 years (OR = 1.4, 95% CI: 1.1–1.8), primiparity (OR = 3.3, 95% CI: 2.7–4.0), para >5 (OR = 3.1, 95% CI: 2.4–4.0), and anemia (OR = 3.3, 95% CI: 2.8–3.9). The risk of pre-eclampsia was inversely increased with education level and prenatal care attendance. The prevalence of placenta previa was 0 (0%) and 55 (3.3%), *P* < 0.001 in pre-eclamptic and control women, respectively. Placenta previa was a significant protective factor of pre-eclampsia (OR = 0.3, 95% CI: 0.1–0.7). Although, the socio-demographic risk factors for pre-eclampsia observed among women at Medani hospital were similar to those found in other settings; placenta previa was associated with decreased incidence of pre-eclampsia.

## Introduction

Pre-eclampsia, is one of the most common complications of pregnancy, it affects approximately 10% of births (Robilland et al., 2003). It is a leading cause of maternal and perinatal mortality worldwide (Duley, 2009). Although, the exact etiology of pre-eclampsia is not yet known, many risk factors have been demonstrated such as low education, primiparity, family history of hypertension, obesity, younger and advanced maternal age, and ethnicity (Conde-Agudelo and Belizán, 2000; Lee et al., 2000; Roberts et al., 2003; Sibai et al., 2005; Adeyinka et al., 2010). Therefore, if women at risk of pre-eclampsia are identified on the basis of epidemiological and clinical risk factors, the awareness can be raised, this will facilitate to make earlier diagnoses and predict which patients are more likely to develop pre-eclampsia and can help to monitor patients as well.

Most of the research on pre-eclampsia was conducted at settings with good resources; few published data exist in low resources settings (Conde-Agudelo and Belizán, 2000). Recently, it has been shown that placenta previa is associated with low frequencies of pre-eclampsia and low maternal blood pressure (Kiondo et al., 2012). However, there are few published studies – with inconsistent findings – on the association between placenta previa and pre-eclampsia. While some studies reported the protective effects, other studies did show any associations, slightly increase in the incidence and significantly elevated incidence pre-eclampsia in placenta previa (Little and Friedman, 1964; Brenner et al., 1978; Newton et al., 1984; Ananth et al., 1997; Hasegawa et al., 2011).

There is an extremely high maternal mortality in Sudan with pre-eclampsia/eclampsia accounting for 4.2% of the obstetric complications and 18.1% of maternal deaths (Leiberman et al., 1991; Ali and Adam, 2011; Ali et al., 2012). The current study was conducted at Medani Maternity Hospital in Central Sudan to investigate the potential risk factors for pre-eclampsia including placenta previa. The data obtained is very useful for the health planners, health providers, and augments the previous research on pre-eclampsia in Sudan (Bakheit et al., 2009, 2010a,b; Elhassan et al., 2009; Adam et al., 2011).

## Materials and Methods

A retrospective case – control study was conducted at labor ward at Medani Maternity Hospital, Central Sudan. Medani Maternity Hospital is a tertiary care hospital for women who receive prenatal care at the hospital as well as for referrals from the other clinics and hospitals. All women with risk factors or obstetric/medical complications are referred to the hospital. However, the referral criteria are not strictly adhered to and many women without any significant complications are allowed to deliver at the hospital. Medical files were reviewed for the patients during the period January 2003 through December 2010. A case was defined as a woman who had given birth and who was diagnosed as being pre-eclamptic. Pre-eclampsia is defined as pregnancy-induced hypertension associated with proteinuria. Pregnancy-induced hypertension is defined as new hypertension with blood pressure of 140 mm Hg systolic or diastolic blood pressure of 90 mm Hg diastolic or greater arising after 20 weeks of gestation in a woman who is normotensive before 20 weeks gestation. Proteinuria is defined as excretion of 300 mg or more of protein in 24 h urine sample or ≥2+ on dipstick. A consecutive control was taken for each case. Controls were parturient women admitted for delivery, without any blood pressure values greater than 139/89 mm Hg or proteinuria recorded in the pregnant health card during the antenatal visits and at the time of delivery. Pregnant women with twins or diabetics were excluded, because these were known predictors for pre-eclampsia (Adeyinka et al., 2010). Placenta previa was diagnosed by ultrasound, confirmed during the cesarean delivery, and documented as such in the medical file.

Data were entered in computer using SPSS for windows version 16.0. Means and proportions were compared by Students’ *t*-test, *X*^2^, and Fisher’s exact tests as appropriate. Univariate and multivariate analyses were performed where pre-eclampsia was a dependent variable and maternal characteristics, age (<20, 20–35, and >35 years), parity (primiparae, 2–5 and para >5), educational levels (illiterate, secondary level ≥ university), and prenatal care (none, once and twice, and >twice), anemia (Hemoglobin <11 gm/dl), and placenta previa as possible influencing factors. Odds ratio and 95% confidence interval were calculated. *P* < 0.05 was regarded as significant.

### Ethics

This was a retrospective study in which the data in the files of the patients were analyzed anonymously and no fresh personal data were required. The study was approved by the local ethical board of the institution.

## Results

During the study period there were 54,339 singleton deliveries among which 1765 women were diagnosed as pre-eclampsia, with the incidence of 3.2%. Out of these women, 1645 had complete data concerning age, parity, education level, prenatal care, placenta previa, and hemoglobin levels and they were included in the final analyses. The excluded 120 women had no placenta previa (excluded by ultrasound) but were not included in the final analyses because of other missing data sets (educational level etc.).

Compared with the controls, significantly higher numbers of pre-eclamptic women had less level of education, prenatal care attendance, and had anemia, Table 1. The mean (SD) age of the pre-eclamptic women was significantly higher than in the controls, 29.0 (6.3) vs. 28.4(6.0) years, *P* = 0.003. There was no case of severe anemia (hemoglobin <7 g/dl).

**Table 1:** **Comparing age, parity, educational levels, prenatal care, anemia, and placenta previa between the cases and controls**.

The variable	Pre-eclamptic women (*n* = 1645)	Controls (*n* = 1645)	*P*
**Age groups in years**
<20	65 (4)	121 (7.4)	<0.001
20–35	1294 (78.7)	1304 (79.3)	0.7
>35	286 (17.4)	220 (13.4)	0.001
**Parity groups**
Primiparae	585 (35.6)	294 (18.0)	<0.001
2–5	724 (44.0)	1217 (74)	<0.001
>5	336 (20.4)	134 (8.0)	<0.001
**Educational level**
Illiterate	451 (27.4)	423 (25.7)	0.2
Secondary	1116 (67.8)	1087 (66.1)	0.2
>Secondary	78 (4.7)	135 (8.2)	<0.001
**Prenatal care visit**
None	409 (24.9)	235 (14.3)	<0.001
One and two	967 (58.8)	835 (50.8)	<0.001
>Two	269 (16.3)	575 (35.0)	<0.001
**Anemia**	1287 (78.2)	738 (44.9)	<0.001
**Placenta previa**	0 (0.0)	55 (3.3)	<0.001

The prevalence of placenta previa was 0 (0%) and 55 (3.3%), *P* < 0.001 in pre-eclamptic and control women, respectively (Table 1).

In logistic regression, the risk factor for pre-eclampsia increased with age, i.e., in comparison to the women with age ranged 20–34 years, younger women (<20 years) were less likely to have pre-eclampsia (OR = 0.2, 95% CI: 0.1–0.3; *P* < 0.001), women with age >35 years were at higher risk for pre-eclampsia (OR = 1.4, 95% CI: 1.1–1.8; *P* = 0.004; Table 2).

**Table 2:** **Risk factors for pre-eclampsia in Medani Hospital using univariate and multivariate analyses**.

The variable	Univariate analysis			Multivariate analysis		
	OR	95% CI	*P*	OR	95% CI	*P*
**Age groups in years**
<20	0.4	0.3–0.7	<0.001	0.2	0.1–0.3	<0.001
20–35	Adam et al. (2011)					
>35	1.3	1.0–1.5	0.006	1.4	1.1–1.8	0.004
**Parity groups**
Primiparae	3.3	2.8–3.9	<0.001	3.3	2.7–4.0	<0.001
2–5	Adam et al. (2011)					
>5	4.2	3.3–5.2	<0.001	3.1	2.4–4.0	<0.001
**Educational level**
Illiterate	1.8	1.3–2.5	<0.001	2.6	2.0–3.5	<0.001
Secondary	1.7	1.3–2.3	<0.001	1.4	1.1–1.8	0.004
>Secondary	Adam et al. (2011)					
**Prenatal care**
None	3.7	3.0–4.6	<0.001	4.2	2.9–6.0	<0.001
Once and twice	2.4	2.0–2.9	<0.001	1.7	1.3–2.1	<0.001
>Twice	Adam et al. (2011)					
**Anemia**	4.4	3.8–5.1	<0.001	3.3	2.8–3.9	<0.001
**Placenta previa**	0.1	0.07–0.3	<0.001	0.3	0.1–0.7	0.006

The other risk factors for pre-eclampsia were primiparity (OR = 3.3, 95% CI: 2.7–4.0; *P* < 0.001) and para >5 (OR = 3.1, 95% CI: 2.4–4.0; *P* < 0.001) and anemia (OR = 3.3, 95% CI: 2.8–3.9 *P* < 0.001). The risk of pre-eclampsia was inversely increased with education level and prenatal care attendance.

Placenta previa was a significant protective factor of pre-eclampsia (OR = 0.3, 95% CI: 0.1–0.7; *P* = 0.006; Table 2).

## Discussion

Perhaps this is the largest data on pre-eclampsia in Africa. In the current study, age (elder age) and parity (primiparae and para >5), low levels of education and low prenatal care attendance were the risk factors for pre-eclampsia. Recently, low education level, primiparity and para ≥5 were shown to be the risk factors for pre-eclampsia in neighboring Uganda (Conde-Agudelo and Belizán, 2000) whereas age and parity were observed to be the risk factors for pre-eclampsia among Latin American and Caribbean women (Adeyinka et al., 2010).

The current study showed that anemic women were three times at higher risk for pre-eclampsia. In eastern Sudan, it has been shown that risk of pre-eclampsia increased only in severe anemia (Hemoglobin <7 gm/dl) and pregnant women with severe anemia were 3.6 times (OR = 3.6, 95% CI: 1.4–9.1) at risk of pre-eclampsia (Ali et al., 2011). The association between pre-eclampsia and anemia might be explained by micronutrient deficiency. Interestingly, it has recently been shown that pregnant women with low levels of vitamin C were at higher risk for pre-eclampsia (Conde-Agudelo and Belizán, 2000). However, it might be difficult to explore if anemia was the cause or effect of pre-eclampsia in the current study and anemia might have been a consequence of the disease process. On the contrary, anemia was reported recently as protective for pre-eclampsia (OR = 0.5, 95% = 0.3–0.9; Kashanian et al., 2011). Yet, in the later study the cut-off was not defined for hemoglobin levels and prevalence of anemia was not significantly different between the cases of pre-eclampsia and controls.

In the present study the incidence of placenta previa was 0 and 3.3% among women with pre-eclampsia and controls, respectively and the risk of pre-eclampsia was reduced by one third among women with placenta previa (OR = 0.3, 95% CI: 0.1–0.7). This goes with the previous studies where it has been shown that the risk of pregnancy-induced hypertension was reduced by half among those with placenta previa (OR = 0.5, 95% CI: 0.3–0.7; Little and Friedman, 1964). Moreover, patients with placenta previa had a third of the risk for hypertensive disorders compared with pregnant women with normally implanted placentae (OR = 0.36, 95% CI: 0.20–0.64; Ananth et al., 1997). Interestingly, recently Hasegawa et al. (2011) have found that not only placenta previa but also low cord insertion were associated with low frequencies of pre-eclampsia and low maternal blood pressure.

It has been suggested that pre-eclampsia originates in the placenta, with inadequate cytotrophoblast invasion, widespread maternal endothelial dysfunction (Brosens, 1977; Wang et al., 2009). During normal early placental development extravillous fetal cytotrophoblasts invade the uterine spiral arteries of the decidua and myometrium and replace the endothelial layer of the maternal spiral arteries, transforming them into large capacitance vessels to provide adequate placental perfusion for the growing fetus. It has been speculated that the blood supply and oxygenation of a placenta implanted in the lower uterine segment are increased compared with those in a placenta implanted in the upper uterine segment because the blood flow to the lower uterine segment is less affected by the pressure of the myometrium (Ananth et al., 1997). The improved blood supply and oxygenation of the placenta previa might explain the decreased incidence of pre-eclampsia.

In summary, the socio-demographic risk factors for pre-eclampsia observed among women at Medani hospital were similar to those found in other settings; placenta previa was associated with decreased incidence of pre-eclampsia.

## Conflict of Interest Statement

The authors declare that the research was conducted in the absence of any commercial or financial relationships that could be construed as a potential conflict of interest.
